# Persistent fatigue in long-COVID is not associated with peripheral inflammatory or cellular stress biomarkers: A cross-sectional controlled study

**DOI:** 10.1016/j.bbih.2026.101226

**Published:** 2026-03-31

**Authors:** Roald Omdal, Ole Bernt Lenning, Grete Jonsson, Jan Terje Kvaløy, Inger Marie Skoie, Geir Sverre Braut, Tore Grimstad

**Affiliations:** a Research Department, Stavanger University Hospital, Stavanger, Norway; b Department of Clinical Science, University of Bergen, Bergen, Norway; c Department of Medical Biochemistry, Stavanger University Hospital, Stavanger, Norway; d Department of Mathematics and Physics, University of Stavanger, Stavanger, Norway; e Department of Dermatology, Stavanger University Hospital, Stavanger, Norway; f Department of Internal Medicine, Stavanger University Hospital, Stavanger, Norway

**Keywords:** Long-COVID, Post-acute sequelae of SARS-CoV-2, Fatigue, Biomarkers, Cytokines, Heat shock proteins, Sickness behavior, Neuroimmune dysfunction

## Abstract

**Background:**

Fatigue persists as a dominant and debilitating phenomenon in long-COVID, yet its underlying biological mechanisms remain unclear. While inflammatory variables tend to normalize within months post-infection, fatigue continues to significantly impact quality of life. Understanding whether specific biomarkers associate with long-COVID fatigue could shed light on pathophysiological mechanisms and potential therapeutic targets.

**Methods:**

In this single-center, cross-sectional controlled study, we enrolled 48 individuals with long-COVID (according to NICE criteria) and 48 age- and sex-matched recovered controls with prior SARS-CoV-2 infection but no persistent symptoms. We carefully excluded all subjects with other diseases or conditions that could influence fatigue levels. Fatigue severity was assessed using three validated instruments: Fatigue Visual Analog Scale (fVAS), Functional Assessment of Chronic Illness Therapy-Fatigue (FACIT-F), and SF-36 vitality subscale. Blood samples were analyzed for pro-inflammatory markers (CRP, TNF-α, IL-6, IL-1β) and biomarkers associated with cellular stress responses and neuroprotection (HSP90α, APOA4, Serpin F1/PEDF, Hemopexin). Anti-nuclear antibodies (ANA) were tested to assess potential autoimmune mechanisms. Depression was assessed using the Hospital Anxiety and Depression Scale, Depression Subscale (HADS-D).

**Results:**

Long-COVID patients demonstrated significantly higher fatigue severity across all instruments compared to recovered controls: fVAS median scores 63 versus 5 (p < 0.001), FACIT-F scores 21.5 versus 49 (p < 0.001), and SF-36 vitality scores 25 versus 72.5 (p < 0.001). Depression scores were also significantly elevated in long-COVID cases. However, none of the measured biomarkers differed significantly between groups: HSP90α, Serpin F1, Hemopexin, APOA4, and CRP showed no differences, while TNF-α and IL-6 showed only tendencies toward higher levels in long-COVID (p = 0.07 and p = 0.07, respectively). IL-1β concentrations were in most cases below the lower limit of detection and were excluded from further analysis. ANA positivity was 10.4% in cases versus 4.2% in controls (p = 0.38) and did not influence fatigue levels. Multivariable regression analysis revealed no significant associations between biomarkers and fatigue severity.

**Conclusions:**

Fatigue in long-COVID represents severe, persistent disability comparable to observations in chronic inflammatory diseases and chronic fatigue syndrome but is not associated with traditional inflammatory biomarkers or cellular stress response proteins measured in peripheral blood. The absence of biomarker associations suggests that long-COVID fatigue may involve more complex mechanisms, potentially including persistent neuro-immune dysregulation, epigenetic changes, or pathophysiological processes not reflected in systemic biomarker concentrations including neurobiological mechanisms such as altered predictive processing and central nervous system–confined neuroinflammation. These findings highlight the need for alternative approaches to understanding and treating long-COVID fatigue beyond conventional inflammatory paradigms.

## Highlights

• Long-COVID fatigue is severe but unrelated to inflammatory biomarkers.
• HSP90α, proinflammatory cytokines, and stress proteins show no association with fatigue severity.
• Recovered controls had fatigue levels comparable to healthy pre-pandemic controls.
• Findings suggest other mechanisms including neuro-immune mechanisms beyond peripheral inflammation.
• Study used rigorous matching and excluded all confounding medical conditions.

## 1 Introduction

Long-COVID is a term used to characterize subjects who, after SARS-CoV-2 infection, experience persisting phenomena including fatigue, headache, decreased memory, brain fog, concentration difficulties, cough, and other neurological and respiratory symptoms and signs. Recent large-scale studies indicate that 4% of children and 10-26% of adults develop long COVID, depending on the computable phenotype used (Mandel et al., 2025). However, controlled estimates accounting for baseline prevalence of these symptoms in uninfected populations suggest an excess attributable risk of approximately 5–6% (RECOVER STUDY). European cohort studies report long-COVID prevalence ranging from 7 to 12% depending on case definition and study population (Thaweethai et al., 2025; de Bruijn et al., 2025).

The mechanisms underlying long-COVID remain unclear but may involve viral persistence, endothelial dysfunction, reactivation of subclinical herpesviruses, dysbiosis, chronic inflammation, altered immune responses, epigenetic changes in hematopoietic and immune cells, and mitochondrial dysfunction in energy metabolism. Recent studies have shown that while many inflammatory parameters tend to normalize within several months to a year post-infection, fatigue persists as a dominant symptom in long-COVID (Atchison et al., 2023; Lenning et al., 2024) even as other associated phenomena become less frequent over time (Fernandez-de-Las-Penas et al., 2022; Mazza et al., 2022). It has a significant impact on patients' quality of life compared to many other issues, with fatigue severity comparable to that seen in chronic inflammatory and autoimmune diseases, cancer, and post-viral fatigue. This underscores the need to explore potential similarities and differences in underlying mechanisms and potential treatment strategies. As research continues, it is becoming increasingly clear that fatigue in long-COVID is not merely a residual effect of the acute infection but a complex, multifaceted phenomenon that significantly impacts patients' quality of life and ability to function in daily activities.

Beyond systemic inflammatory pathways, purely neurobiological mechanisms may also contribute to long-COVID fatigue. Isolated neuroinflammation confined to the central nervous system and disrupted predictive brain processing - wherein the brain's internal predictive model becomes misaligned with actual bodily signals—represent plausible alternative mechanisms (Moen et al., 2025; Greenhouse-Tucknott et al., 2022). These neurobiological frameworks are particularly relevant given that fatigue in long-COVID often persists despite normalization of peripheral inflammatory markers, suggesting that central nervous system processes may play a significant role in symptom persistence.

Fatigue can be defined as "an overwhelming sense of tiredness, lack of energy, and feeling of exhaustion (Krupp and Pollina, 1996). In chronic inflammatory conditions such as rheumatoid arthritis, systemic lupus erythematosus, and inflammatory bowel disease, fatigue may result from sustained activation of the innate immune system. Proinflammatory cytokines such as interleukin-1β (IL-1β), tumor necrosis factor-alpha (TNF-α), and interleukin-6 (IL-6) are frequently elevated in these conditions, leading to systemic inflammation (Bhol et al., 2024).

The concept of "sickness behavior" provides a unifying framework for understanding fatigue in such conditions. Sickness behavior refers to a highly coordinated adaptive response comprising behavioral and physiological changes, including fatigue, anorexia, reduced activity, and cognitive impairment, that is mediated by the immune system in response to infection or inflammation (Dantzer, 2001). This adaptive response is mediated by cytokines and other biomolecules that signal to the brain.

An additional objective was to assess whether fatigue levels in recovered controls were comparable to those of healthy individuals from a pre-pandemic population (Skoie et al., 2017).

## 2 Materials and methods

### 2.1 Study design and participants

In this single-center case-control study, individuals aged 16-80 years with prior SARS-CoV-2 infection were considered for inclusion. The inclusion criterion was a verified infection confirmed by PCR testing or SARS-CoV-2 antigen self-testing. Participants were classified as long-COVID cases if they fulfilled NICE criteria for long-COVID (National Institute for Health and Care Excellence: Clinical Guidelines, 2024), which require: (a) symptoms developing during or after SARS-CoV-2 infection consistent with COVID-19, (b) symptom persistence for more than 12 weeks from symptom onset, and (c) symptoms not explained by an alternative diagnosis. Case ascertainment required physician interview documenting persistent symptoms including fatigue, dyspnea, cognitive dysfunction, headache, and/or functional impairment. Symptom severity and duration were documented during the clinical interview; no formal cutoff scores were applied, and diagnosis was based on clinical judgment according to the NICE framework. Exclusion criteria included other conditions possibly associated with fatigue, such as concomitant autoimmune or chronic inflammatory diseases, cancer, anemia (hemoglobin <100 g/L), and untreated hypothyroidism, inability to provide informed consent or adhere to the study protocol, and cases deemed inappropriate for participation by the study physician.

Patients were recruited within the Stavanger community area. Eligible patients identified by general practitioners were contacted by the study physician and provided written informed consent prior to inclusion. Participants considered to have long-COVID syndrome according to the NICE criteria were assigned as cases, while patients with prior SARS-CoV-2 infection and no signs of long-COVID were assigned as recovered controls. These groups were age- and sex-matched.

Of the initial 52 referred individuals with suspected long-COVID, four were excluded due to: vaccine reaction (n = 1), Graves' disease (n = 1), thyroid cancer (n = 1), and undiagnosed neurological condition (n = 1), ultimately resulting in 48 participants being included in the final analysis. Eligible recovered controls were identified through general practitioner networks in the same community area and recruited between January 2022 and November 2023. Matching was performed by the study physician using frequency matching on age (±5 years) and sex. Time since infection was not used as a formal matching criterion; however, median time since infection was similar between groups (65.5 weeks in long-COVID cases vs. 72.5 weeks in recovered controls, p = 0.54), reducing confounding from temporal effects on biomarker concentrations. In the recovered group, 12 subjects were excluded due to inadequate age- and sex-matching with cases, anemia (n = 1) and hypothyroidism (n = 1). The final study sample consisted of 48 participants with long-COVID and 48 age- and sex-matched recovered controls, totaling 96 participants for biomarker analysis (Table 1).

**Table 1:** Characteristics of 48 patients with long-COVID and 48 recovered controls. Median and interquartile ranges (IQR) are given, except age (mean 95% CI).

	All patients (n = 96)	Long-COVID (LC) cases (n = 48)	Recovered Controls (RC) (n = 48)	Healthy control subjects (HCS) (n = 40)	*p*-value (LC vs. RC) (n = 48 vs. n = 48)	*p-value* (RC vs. HCS) (n = 40 vs. n = 40)
**Age, years (mean, 95%CI)**	46.7 (44.2-49.2)	46.9 (43.4-50.3)	46.6 (46.6-42.9)			
**Sex, female/male, n (%)**	82 (85.4)/14 (14.6)	41 (84.4)/7 (14.6)	41 (84.4)/7 (14.6)			
**Time since COVID-19 diagnosis, weeks (median [IQR])**	69.0 (46.25-102.25)	65.5 (42.5-102.25)	72.5 (51.5-104)		0.54	
**HSP90α ng/mL**		9.3 (7.8-11.0)	10.1 (8.6-11.4)		0.20	
**Serpin F1 μg/mL**		13.7 (11.8-17.3)	13.0 (10.4-16.3)		0.65	
**HPX μg/mL**		620 (564-702)	610 (556-658)		0.32	
**APOA4 μg/mL**		3.6 (3.2-4.1)	3.6 (3.3-4.0)		0.71	
**CRP mg/L**		1.0 (0-3.0)	0 (0-2.0)		0.32	
**TNF- α fg/mL**		272 (208-371)	244 (206-298)		0.07	
**IL-6 fg/mL**		1528 (1010-2312)	1083 (830-2106)		0.07	
**ANA (positive/negative), n**		5/43	2/46		0.38	
**fVAS**		63 (56-75)	5 (0-16)	11.5 (3-20)	<0.001	0.11
**FACIT-F**		21.5 (14-28)	49 (47-51)	N.A.	<0.001	N.A
**SF-36 VS**		25 (10-35)	72.5 (61-80)	70 (65-80)	<0.001	0.68
**HADS-D**		7 (4-9)	0 (0-1)	1 (0-3)	<0.001	<0.001
**SARS-CoV-2 diagnostic tests, n (%)**^a^**:**						
**PCR**		21 (43.7)	48 (100)			
**Antigen Self-test**		40 (83.3)	4 (8.3)			

Abbreviations: HSP, Heat Shock Protein; HPX, Hemopexin; APOA, Apolipoprotein-A; CRP, C-Reactive Protein; TNF, Tumor Necrosis Factor; IL, Interleukin; ANA, Antinuclear Antibody, fVAS, fatigue Visual Analog Scale; FACIT-F, Functional Assessment of Chronic Illness Therapy-Fatigue; SF-36 VS, Short Form-36 Vitality Subscale, HADS-D, Hospital Anxiety and Depression Scale, Depression subscale; N.A., not applicable.

a 13 (27.1%) of cases and 4 (8.3%) of recovered controls performed two diagnostic tests.

Demographic and clinical data were recorded, blood samples were collected, and selected health questionnaires were completed under supervision before blood sampling in all study participants.

### 2.2 Fatigue and health assessment instruments

Three widely used and internationally accepted generic and uni-dimensional fatigue instruments, all with established internal consistency and reliability were used. The fatigue questionnaires were handed out to study participants during the study visit and the study physician was present and available for questions during assessments.

fVAS: The fatigue Visual Analog Scale instrument (Wolfe et al., 1996). This instrument consists of a 100 mm line with vertical anchors at each end, indicating “no fatigue” at the left end (0 mm) and “fatigue as bad as it can be” at the right end (100 mm). The average experienced fatigue during the last two weeks is then marked on the line by the patient, and higher scores indicates more fatigue.

FACIT-F: Functional Assessment of Chronic Illness Therapy (Cella et al., 2005). This measure contains 40 items, each rated on a Likert scale from 0 to 4, where the total score is 0-52, with higher scores indicating less fatigue during the last 7 days.

SF-36 Vitality Subscale: Medical Outcome Study Short Form 36 vitality dimension for evaluation of fatigue severity (Ware and Sherbourne, 1992). This instrument is a SF-36 subscale, containing four questions assessing fatigue and energy during the last 4 weeks. The total score is 0-100, with higher levels corresponding to less fatigue (more “vitality”).

Clinical depression was assessed with the Hospital Anxiety and Depression Scale (HADS-D) (Zigmond and Snaith, 1983). This measure consists of 7 items, rated from 0 to 3 on a Likert scale. A higher score is indicative of more depressive mood.

### 2.3 Laboratory tests

Blood samples were drawn by venous puncture, and routine hematological and biochemical analyses performed at the hospital laboratory. Anti-nuclear antibodies (ANA) were analyzed at Haukeland University Hospital in Bergen, using the BioPlex 2200 ANA Screen on the Bio-Rad BioPlex 2200 System (Bio-Rad, Hercules, CA, USA). This test includes quantitative detection of dsDNA antibody and semi-quantitative detection of antibodies against Chromatin, Ribosomal P, SS-A, SS-B, Sm, SmRNP, RNP, Scl-70, Jo-1, and Centromere B.

For exploratory biomarkers, venous blood collected in serum tubes was allowed to clot for 30 min at room temperature, and thereafter centrifuged (15 min at 2500×*g* at 4 °C), aliquoted and stored at − 80 °C until analysis. Blood collected in EDTA tubes was centrifuged, aliquoted and plasma stored as described for serum within 30 min of sampling.

Heat Shock Protein 90α (HSP90α), Hemopexin (HPX), Apolipoprotein A4 (APOA4) and Human Serpin F1/PEDF in serum samples were analyzed by commercially available ELISA-kits from Enzo Life Sciences, Farmingdale, NY, USA (HSP90α), CusaBio, Houston, TX, USA (HPX and APOA4) and Biotechne R&D, Minneapolis, MN, USA (Serpin F1). Tumor Necrosis Factor-α (TNF-α), Interleukin-6 (IL-6) and Interleukin-1β (IL-1β) in EDTA-plasma were analyzed by Meso® QuickPlex SQ 120 Imager (Meso Scale Diagnostics, Rockville, MD, USA) using the MSD S-PLEX® Platform for the proinflammatory panel 1 (human) kit (Meso Scale Diagnostics, Rockville, MD, USA). All analyses were carried out in accordance with the manufacturer's protocols.

The biomarker panel was selected based on prior evidence linking these molecules to fatigue in chronic inflammatory and post-viral conditions as stated in the introduction. ANA tests were analyzed descriptively; including all measured biomarkers in multivariable models would exceed our sample size capacity and increase the risk of Type I error without strong theoretical justification.

### 2.4 Statistical analysis

For continuous data, normal distribution was tested using the Shapiro-Wilk test. For continuous variables, the Wilcoxon signed rank test (when non-normal distribution) or paired *t*-test (when normal distribution, only SF-36 VS) was used to test for differences in distribution between two paired groups, and McNemar's exact test for categorical variables. Univariable regression was initially performed in the long-COVID group using, respectively, fVAS, FACIT-F, and SF-36 vitality subscale scores as dependent variables, with sex, age, HSP90α, CRP, TNF-α and IL-6 as independent variables. Separate multivariable regression models were then fitted with the same dependent variables and age, sex, and independent variables yielding p ≤ 0.2 in the univariable analysis.

With 48 patients with long-COVID and matched recovered controls there was a power of 99.9% for detecting a difference in fVAS of at least 20, using the Wilcoxon signed rank test and assuming a standard deviation of the difference in fVAS scores of 20.

In terms of the linear regression analysis, this sample size implies a power of 75% to detect a medium effect size and a power of 98% to detect a large effect size in the univariable linear regression models, using Cohen's (Cohen, 1988) definition of medium (f^2^ = 0.15, r^2^ = 0.13) and large (f^2^ = 0.35, r^2^ = 0.26) effect size.”

The IBM SPSS Statistics 29 software was used, and p-value of <0.05 was considered significant. Due to the exploratory nature of this study, corrections for multiple comparisons were not performed and p-values should be interpreted as explorative.

## 3 Results

### 3.1 Descriptives

The mean (95% CI) age of participants in these two groups was 46.7 (44.2-49.2) years for all; 46.9 (43.4-50.3) years in long-COVID cases and 46.6 (42.9-50.2) in recovered controls, respectively. In this sample (n = 96), 82 of 96 (85.4%) were females, whereas 41 of 48 (84.4%) were females in each of the two subgroups (n = 48 in each). Time since diagnosis (i.e. time since infection) was median (interquartile range) 69 (46-102.-) weeks for all; 65.5 (43-102) in long-COVID cases and 72.5 (52-104) in recovered controls, p = 0.54 (Table 1).

### 3.2 Exploratory biomarkers

Biomarker concentrations in 48 long-COVID cases versus 48 recovered controls were not significantly different for the following variables: Heat Shock Protein 90α (HSP90α), Serpin F1, Hemopexin (HPX), Apolipoprotein A4 (APOA4), C-reactive protein (CRP), Tumor Necrosis Factor-α (TNF-α) and Interleukin-6 (IL-6) (Table 1). IL-1β concentrations were predominantly below the lower limit of detection and were therefore excluded from analysis. Thus, no statistically significant differences were observed between long-COVID cases and recovered controls.

### 3.3 Anti-nuclear antibody (ANA) testing

ANA was low-titered positive in 5 of 48 long-COVID cases (10.4%) and in 2 of 48 recovered controls (4.2%), with no significant difference between groups (p = 0.38), Table 1.

Since TNF-α and IL-6 levels showed a tendency toward differences between long-COVID cases and recovered controls (p = 0.07 and p = 0.07, respectively), we investigated whether this could be attributed to inflammatory activity associated with positive ANA status. However, subgroup analysis revealed no significant differences between ANA-positive and ANA-negative participants, Supplementary Table 1.

### 3.4 Fatigue

**Fig. 1:**
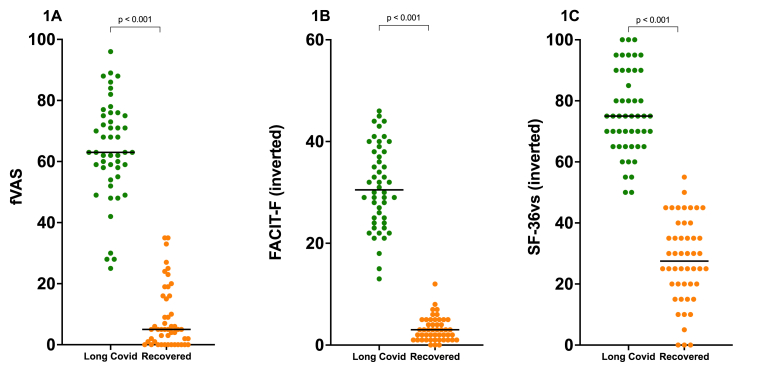
Fatigue severity in long-COVID cases versus recovered controls. Comparison of fatigue scores using three validated instruments: (A) Fatigue Visual Analog Scale (fVAS), (B) Functional Assessment of Chronic Illness Therapy-Fatigue (FACIT-F) inverted scores, and (C) SF-36 vitality subscale inverted scores. All comparisons showed p < 0.001. Higher scores indicate greater fatigue severity.

The fVAS, FACIT-F, and SF-36 vitality subscale scores differed significantly between long-COVID cases and recovered controls, Table 1, Fig. 1. Inverted scores for FACIT-F and SF-36 vitality subscale are presented for easier interpretation, with higher values indicating more fatigue severity (Fig. 1).

### 3.5 Hospital Anxiety and Depression Scale (HADS)

The HADS-D scores were significantly higher in cases with long-COVID as compared with recovered controls, showing median (interquartile range) 7 (Atchison et al., 2023; Lenning et al., 2024; Fernandez-de-Las-Penas et al., 2022; Mazza et al., 2022; Moen et al., 2025; Greenhouse-Tucknott et al., 2022) vs. 0 (0-1), p < 0.001 (Supplementary Fig. 1).

### 3.6 Healthy control subjects

We aimed to determine whether the level of fatigue in the recovered controls was comparable to that of healthy individuals. To address this, we utilized data from formally healthy subjects obtained in a previous pre-pandemic study (Skoie et al., 2017). Fatigue, assessed by both the fVAS and SF-36 vitality subscale, and depression, assessed by HADS-D, were compared between the recovered controls and age- and gender-matched healthy controls (n = 40 per group). Group characteristics are presented in Supplementary Table 1. There were no significant differences in fVAS or SF-36 vitality subscale scores between recovered and pre-pandemic healthy controls. However, HADS-D depression scores were significantly lower in the recovered controls than in the pre-pandemic healthy control subjects (0 [0–1.0] vs. 1 [0–3], p = 0.001 (Supplementary Fig. 2).

### 3.7 Regression analysis

In univariable analysis, age tended to be negatively associated with FACIT-F scores (p = 0.06), but no significant associations occurred between the selected variables and fatigue (Table 2). Also, multivariable regression models, applying fVAS, FACIT-F, SF-36 vitality subscale and HADS-D scores, respectively as dependent variables, revealed no significant associations (Table 3).

**Table 2:** Univariable regression analysis in 48 long-COVID cases, applying fVAS, FACIT-F, SF-36VS and HADS-D scores as dependent variables in separate models.

Variable	fVAS			FACIT-F			SF-36VS			HADS-D		
	β	95% CI	p-value	β	95% CI	p-value	β	95% CI	p-value	β	95% CI	p-value
Age	−0.12	−0.56-0.24	0.43	0.28	−0.0-0.39	0.06	0.11	−0.21-0.46	0.46	0.07	−0.07-0.11	0.67
Sex	−0.25	−24.6-1.67	0.09	0.08	−5.09-8.58	0.61	0.08	−8.32-14.24	0.60	−0.20	−4.87-1.02	0.20
HSP90 ng/ml	0.22	−0.31-2.23	0.14	−0.06	−0.80-0.52	0.67	−0.07	−1.33-0.84	0.66	0.17	−0.12-0.45	0.25
CRP (mg/l)	0.12	−0.87-2.03	0.43	−0.12	−1.04-0.43	0.41	−0.04	−1.37-1.06	0.80	0.03	−0.29-0.35	0.85
TNF-α (fg/ml)	−0.02	−0.04-0.03	0.92	0.20	−0.01-0.03	0.17	0.16	−0.01-0.05	0.26	−0.01	−0.01-0.01	0.97
IL-6 (fg/ml)	−0.03	−0.002-0.002	0.86	0.1	−0.001-0.001	0.97	−0.10	−0.002-0.001	0.51	0.24	0.00-0.001	0.12

Standardized β -values are reported. i.e. the impact of a one-standard deviation increase in the corresponding independent variable.

Abbreviations: fVAS, fatigue Visual Analog Scale; FACIT-F, Functional Assessment of Chronic Illness Therapy-Fatigue; SF-36VS, Medical Outcomes Study 36-Item Short-Form Health Survey, Vitality Subscale; HADS-D, The Hospital Anxiety and Depression Scale, Depression Subscale; HSP, Heat Shock Protein; CRP, C-Reactive Protein; TNF, Tumor Necrosis Factor; IL, Interleukin.

**Table 3:** Multivariable regression models in 48 long-COVID cases, applying fVAS, FACIT-F, SF-36VS and HADS-D scores as dependent variables in separate models.

Variable	fVAS			FACIT-F			SF-36VS			HADS-D		
	β	95% CI	p-value	β	95% CI	p-value	β	95% CI	p-value	β	95% CI	p-value
Age	−0.08	−0.50-0.28	0.59	0.27	−0.02-0.39	0.07				−0.11	−0.1-0.09	0.95
Sex	−0.27	−25.31-0.83	0.07	0.06	−5.33-8.20	0.67				−0.22	−5.05-0.81	0.15
HSP90 ng/ml	0.24	−0.22-2.30	0.10	−0.04	−0.75-0.56	0.77						
IL-6 (fg/ml)										0.26	0.00-0.001	0.11
Total model:	R^2^ = 0.123, p = 0.11			R^2^ = 0.08, p = 0.28			N.A.			R^2^ = 0.10, p = 0.21		

Standardized β -values are reported. i.e. the impact of a one-standard deviation increase in the corresponding independent variable.

Abbreviations: fVAS, fatigue Visual Analog Scale; FACIT-F, Functional Assessment of Chronic Illness Therapy-Fatigue; SF-36VS, Medical Outcomes Study 36-Item Short-Form Health Survey, Vitality Subscale; HADS-D, The Hospital Anxiety and Depression Scale, Depression Subscale; HSP, Heat Shock Protein; CRP, C-Reactive Protein; TNF, Tumor Necrosis Factor; IL, Interleukin.

## 4 Discussion

### 4.1 Severity and prevalence of fatigue in Long-COVID versus recovered controls

Despite profound fatigue in long-COVID patients, evident across all three validated instruments at a median of 69 weeks after infection, no significant differences were observed between long-COVID cases and recovered controls in any measured inflammatory markers (CRP, TNF-α, IL-6) or cellular stress response proteins (HSP90, Serpin F1, Hemopexin, APOA4). Notably, multivariable regression analysis revealed no associations between any biomarkers and fatigue severity. This discordance between clinical symptom severity and peripheral biomarker profiles suggests that long-COVID fatigue may involve pathophysiological mechanisms not captured by traditional inflammatory paradigms.

The FACIT-F and SF-36 vitality subscale scores demonstrated profound fatigue severity in long-COVID patients that is comparable to levels reported in chronic inflammatory diseases (rheumatoid arthritis (Valencia-Muntala et al., 2024), cancer (Wagner and Cella, 2004), and myalgic encephalomyelitis/chronic fatigue syndrome (Park et al., 2024)).

These findings align with recent large-scale studies showing that while many post-COVID phenomena tend to resolve over time, fatigue persists as the most prominent and enduring complaint (Fernandez-de-Las-Penas et al., 2022; Mazza et al., 2022). The severity observed in our cohort, with a median time since diagnosis of approximately 69 months, underscores that long-COVID fatigue is not merely a transient post-viral phenomenon but represents a persistent abnormal condition with major impact also in what appears as the chronic phase of long-COVID. This is consistent with Fernandez-de-Las-Penas et al.'s findings that post-COVID-19 symptoms, particularly fatigue, persist even 2 years after initial infection (Fernandez-de-Las-Penas et al., 2022).

### 4.2 Fatigue severity in recovered controls versus healthy pre-pandemic controls

Fatigue levels in recovered controls were comparable to those of healthy pre-pandemic controls (Skoie et al., 2017) suggesting that this group represents an appropriate comparator with fatigue levels within the expected population range (Supplementary Table 2). The observed difference in HADS-D scores between these groups was unexpected and may reflect methodological differences between studies (Supplementary Fig. 2).

### 4.3 Associations between fatigue severity and candidate biomarkers in Long-COVID

Despite selecting biomarkers based on established associations with fatigue in chronic inflammatory conditions and previous long-COVID research (Cho et al., 2013; Tasson et al., 2021; Moss et al., 1999; Druce et al., 2015; Strawbridge et al., 2019; Bardsen et al., 2016; Grimstad et al., 2020; Kvivik et al., 2023; Diniz et al., 2016; Larssen et al., 2019; Baraniuk et al., 2005; Del Valle et al., 2020), we found no significant associations between any of the measured biomarkers and fatigue severity in our long-COVID cohort. Univariable and multivariable regression analysis failed to identify significant predictors of fatigue across all three fatigue scales.

Several factors may explain these negative findings. First, the heterogeneous nature of long-COVID may require different biomarker profiles in subgroups of patients (Mandel et al., 2025). Second, fatigue in long-COVID may be mediated by mechanisms not captured by the traditional inflammatory and stress response markers we examined. The concept of "sickness behavior" as an adaptive response to infection or inflammation may be more complex in long-COVID, potentially involving persistent dysregulation of neuro-immune pathways that are not reflected in peripheral biomarker concentrations (Dantzer, 2001) or possibly be due to epigenetic changes that are not reflected in inflammatory biomolecules but involve adaptive behavioral phenomena (Apostolou and Rosen, 2024).

The tendency toward higher TNF-α and IL-6 levels in long-COVID cases (p = 0.07 and p = 0.07, respectively) could suggest subtle inflammatory activity, but these differences were not sufficient to reach statistical significance and did not correlate with fatigue severity These results are consistent with studies showing that while acute COVID-19 is characterized by pronounced inflammatory responses, long-COVID may involve more subtle inflammatory processes not readily detectable in peripheral blood, and possibly only operative in long-COVID subtypes (Mandel et al., 2025).

### 4.4 Age, sex, and other demographic associations

Age emerged as a close to significant predictor of fatigue severity in univariable analysis specifically in the FACIT-F model (β = 0.28, p = 0.06), with older individuals experiencing more severe fatigue. This finding aligns with clinical observations that older adults may have more persistent symptoms following viral infections and is consistent with epidemiological data showing age as a risk factor for long-COVID development (Mandel et al., 2025). Age is not consistently associated with fatigue in other conditions and could be related to use of different fatigue instruments, selection of patients or other factors.

Likewise, female sex showed a tendency toward lower fVAS score (β = −0.25, p = 0.09) suggesting potential sex-related differences in fatigue expression or reporting, though this did not reach statistical significance.

### 4.5 ANA results and autoimmune implications

We carefully excluded all subjects from our cohorts with known autoimmune conditions that could influence fatigue. The prevalence of positive ANA in long-COVID cases (10.4%) was not significantly different from recovered controls (4.2%), and ANA positivity did not influence biomarker levels or fatigue severity. This finding suggests that classic autoimmune mechanisms, as detected by conventional ANA testing, are not a major driver of long-COVID fatigue in our sample. However, this does not exclude more subtle autoimmune processes or the possibility that other autoantibodies not included in our screening panel may be involved.

### 4.6 Study limitations, strengths and clinical implications

Study limitations: Several limitations should be acknowledged. The cross-sectional design cannot establish causality or temporal relationships between biomarkers and symptoms. Additionally, our biomarker panel, while comprehensive, may not have captured specific biological pathophysiological mechanisms underlying long-COVID fatigue. Furthermore, the sample size of 48 long-COVID cases, while adequately powered for between-group comparisons of fatigue, may have been insufficient to detect small-to-moderate associations between individual biomarkers and fatigue severity in the regression analyses. The absence of significant biomarker–fatigue associations should therefore be interpreted with caution, and larger studies are needed to exclude clinically meaningful but modest associations.

Study strengths: This study employed rigorous design with laboratory-verified SARS-CoV-2 infection and physician assessment. The case-control design age- and sex-matched 48 long-COVID patients with 48 recovered controls (both with prior infection) and systematically excluded comorbidities that could influence fatigue. Comprehensive fatigue assessment used three validated instruments. The inclusion of a third comparator group of 40 healthy pre-pandemic controls demonstrated that fatigue levels in recovered controls were comparable to those of the general population, supporting the validity of this group as a non-fatigued comparator.

Clinical implications: The absence of significant biomarker associations does not diminish the clinical reality of severe fatigue in long-COVID patients. Rather, it highlights the need for alternative approaches to understanding and treating this debilitating symptom. Future research should explore neuroinflammatory processes and epigenetic changes that may be linked to long-COVID pathophysiology (Calzari et al., 2024; de Vega et al., 2014; Braekke et al., 2016).

## CRediT authorship contribution statement

**Roald Omdal:** Writing – review & editing, Writing – original draft, Resources, Project administration, Methodology, Funding acquisition, Formal analysis, Data curation, Conceptualization. **Ole Bernt Lenning:** Writing – review & editing, Writing – original draft, Investigation, Formal analysis, Data curation, Conceptualization. **Grete Jonsson:** Writing – review & editing, Writing – original draft, Validation, Methodology, Investigation, Formal analysis, Data curation. **Jan Terje Kvaløy:** Writing – review & editing, Writing – original draft, Validation, Supervision, Methodology, Formal analysis, Data curation. **Inger Marie Skoie:** Writing – review & editing, Writing – original draft, Resources, Investigation, Formal analysis, Data curation. **Geir Sverre Braut:** Writing – review & editing, Writing – original draft, Validation, Methodology, Data curation, Conceptualization. **Tore Grimstad:** Writing – review & editing, Writing – original draft, Visualization, Validation, Methodology, Formal analysis, Data curation, Conceptualization.

## Funding

This work was supported by 10.13039/100009471Foundation Dam (Stiftelsen Dam) (grant number #SDAM_HEL45920) and The Norwegian Brain Council.

## Declaration of competing interest

The authors declare the following financial interests/personal relationships which may be considered as potential competing interests:Tore Grimstad reports a relationship with TG has received unrestricted research grants from AbbVie, Tillotts Pharma AB, has served as speaker for AbbVie, Ferring Pharmaceuticals, Takeda AS, and has been advisory board member for Takeda AS, Johnson & Johnson, Tillotts Pharma AB and Galapagos. that includes: board membership and speaking and lecture fees. If there are other authors, they declare that they have no known competing financial interests or personal relationships that could have appeared to influence the work reported in this paper.IMS has served as speaker for UCB and Leo Pharma.

## Data Availability

Data will be made available on request.

## Appendix A Supplementary data

Multimedia component 2 Multimedia component 2

Multimedia component 3 Supplementary Figure 1. Hospital Anxiety and Depression Scale, Depression Subscale (HADS-D) scores in long-COVID cases versus recovered controls (n = 48 per group. Median (range) scores were 7 (Mandel et al., 2025; Thaweethai et al., 2025; de Bruijn et al., 2025; Atchison et al., 2023; Lenning et al., 2024; Fernandez-de-Las-Penas et al., 2022; Mazza et al., 2022; Moen et al., 2025; Greenhouse-Tucknott et al., 2022; Krupp and Pollina, 1996; Bhol et al., 2024; Dantzer, 2001; Skoie et al., 2017; National Institute for Health and Care Excellence: Clinical Guidelines, 2024; Wolfe et al., 1996; Cella et al., 2005; Ware and Sherbourne, 1992) vs. 0 (0-3), p < 0.001. Multimedia component 3

Multimedia component 4 Supplementary Figure 2. Comparison of HADS-D scores among recovered controls, and healthy pre-pandemic controls (n = 40 per group), with significantly higher HADS-D scores in healthy controls (p = 0.001). Multimedia component 4
