# Supplementary material for: Persistent fatigue in long-COVID is not associated with peripheral inflammatory or cellular stress biomarkers: A cross-sectional controlled study
Source: Brain Behav Immun Health. 2026 Mar 31;54:101226. doi: 10.1016/j.bbih.2026.101226 (PMC13087645; doi:10.1016/j.bbih.2026.101226)
Supplement: Multimedia component 1 [file mmc1.docx]

|  | **Recovered controls**  **(n=40)** | **Healthy pre-pandemic control subjects (n=40)** | **p-value** |
| --- | --- | --- | --- |
| Age (years) | 46.5 (19-73) | 46.5 (20-70) | 0.85 |
| Sex, female/male, n (%) | 33/7 (83/17) | 33/7 (83/17) | 1.0 |
| fVAS | 5 (0-35) | 11.5 (0-54) | 0.11 |
| SF-36VS | 70 (50-100) | 75 (35-100) | 0.68 |
| HADS-D | 0 (0-3) | 1 (0-10) | <0.001 |

**Supplementary Table 1**

**Characteristics of 80 subjects in two separate groups; recovered controls, and pre-pandemic healthy control subjects**

**Abbreviations**: fVAS, fatigue Visual Analog Scale; SF-36VS, Medical Outcomes Study 36-Item Short-Form Health Survey, Vitality Subscale; HADS-D, The Hospital Anxiety and Depression Scale, Depression Subscale
