# Supplementary material for: Persistent fatigue in long-COVID is not associated with peripheral inflammatory or cellular stress biomarkers: A cross-sectional controlled study
Source: Brain Behav Immun Health. 2026 Mar 31;54:101226. doi: 10.1016/j.bbih.2026.101226 (PMC13087645; doi:10.1016/j.bbih.2026.101226)
Supplement: Multimedia component 2 [file mmc2.docx]

**Supplementary Table 2**

**Univariable Regression Analysis in 48 Long-COVID Cases, Applying fVAS, FACIT-F, SF-36VS, and HADS-D Scores as Dependent Variables in Separate Models**

| **Variable** | **fVAS** | | **FACIT-F** | | **SF-36VS** | | **HADS-D** | |
| --- | --- | --- | --- | --- | --- | --- | --- | --- |
|  | β | p-value | β | p-value | β | p-value | β | p-value |
| Age | -0.12 | 0.43 | 0.28 | 0.06 | 0.11 | 0.46 | 0.07 | 0.67 |
| Sex | -0.25 | 0.09 | 0.08 | 0.61 | 0.08 | 0.60 | -0.20 | 0.20 |
| HSP90 (ng/mL) | 0.22 | 0.14 | -0.06 | 0.67 | -0.07 | 0.66 | 0.17 | 0.25 |
| CRP (mg/L) | 0.12 | 0.43 | -0.12 | 0.41 | -0.04 | 0.80 | 0.03 | 0.85 |
| TNF-α (fg/mL) | -0.02 | 0.92 | 0.20 | 0.17 | 0.16 | 0.26 | -0.1 | 0.97 |
| IL-6 (fg/mL) | -0.03 | 0.86 | 0.1 | 0.97 | -0.10 | 0.51 | 0.24 | 0.12 |

Standardized β -values are reported. i.e. the impact of a one-standard-deviation increase in the corresponding independent variable.

Abbreviations: fVAS, fatigue Visual Analog Scale; FACIT-F, Functional Assessment of Chronic Illness Therapy-Fatigue; SF-36VS, Medical Outcomes Study 36-Item Short-Form Health Survey, Vitality Subscale; HADS-D, The Hospital Anxiety and Depression Scale, Depression Subscale; HSP, Heat Shock Protein; CRP, C-Reactive Protein; TNF, Tumor Necrosis Factor; IL, Interleukin.
