# Supplementary figures and images for: Persistent fatigue in long-COVID is not associated with peripheral inflammatory or cellular stress biomarkers: A cross-sectional controlled study
Source: Brain Behav Immun Health. 2026 Mar 31;54:101226. doi: 10.1016/j.bbih.2026.101226 (PMC13087645; doi:10.1016/j.bbih.2026.101226)

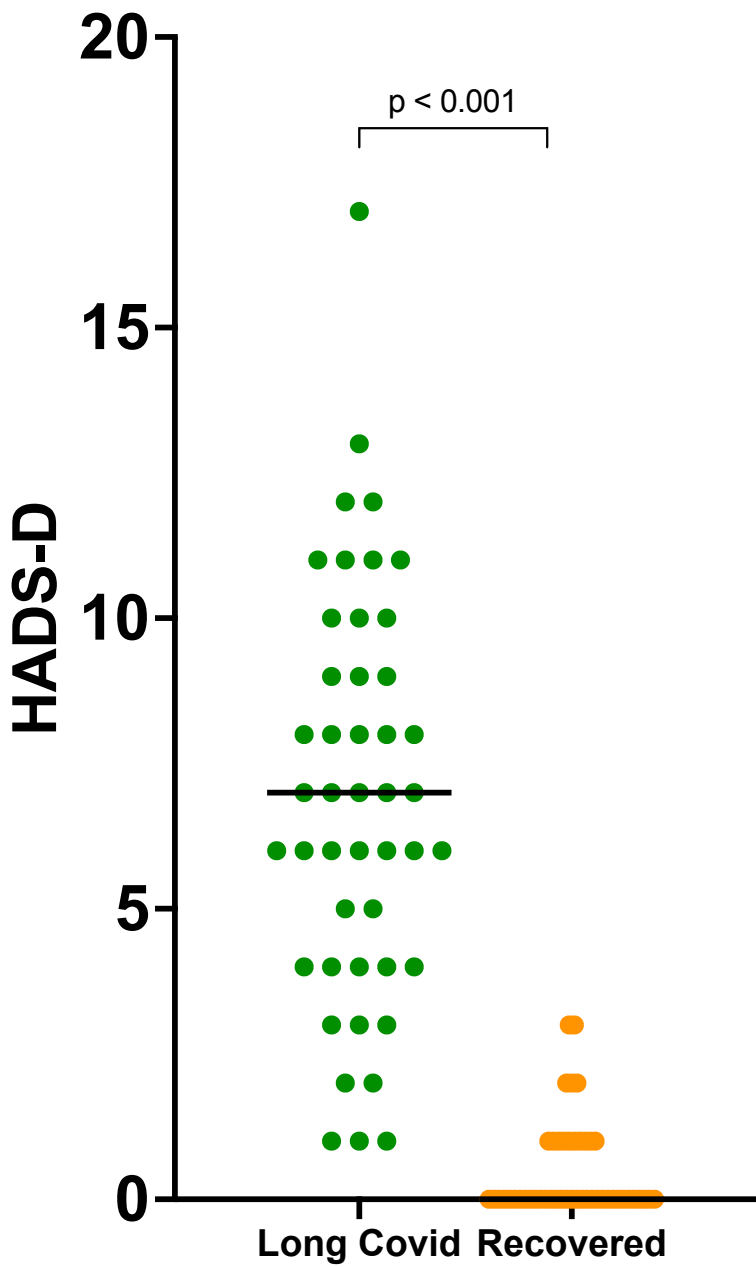

Supplement: Multimedia component 3 — Supplementary Figure 1. Hospital Anxiety and Depression Scale, Depression Subscale (HADS-D) scores in long-COVID cases versus recovered controls (n = 48 per group. Median (range) scores were 7 (Mandel et al., 2025; Thaweethai et al., 2025; de Bruijn et al., 2025; Atchison et al., 2023; Lenning et al., 2024; Fernandez-de-Las-Penas et al., 2022; Mazza et al., 2022; Moen et al., 2025; Greenhouse-Tucknott et al., 2022; Krupp and Pollina, 1996; Bhol et al., 2024; Dantzer, 2001; Skoie et al., 2017; National Institute for Health and Care Excellence: Clinical Guidelines, 2024; Wolfe et al., 1996; Cella et al., 2005; Ware and Sherbourne, 1992) vs. 0 (0-3), p < 0.001. [file mmc3.pdf]

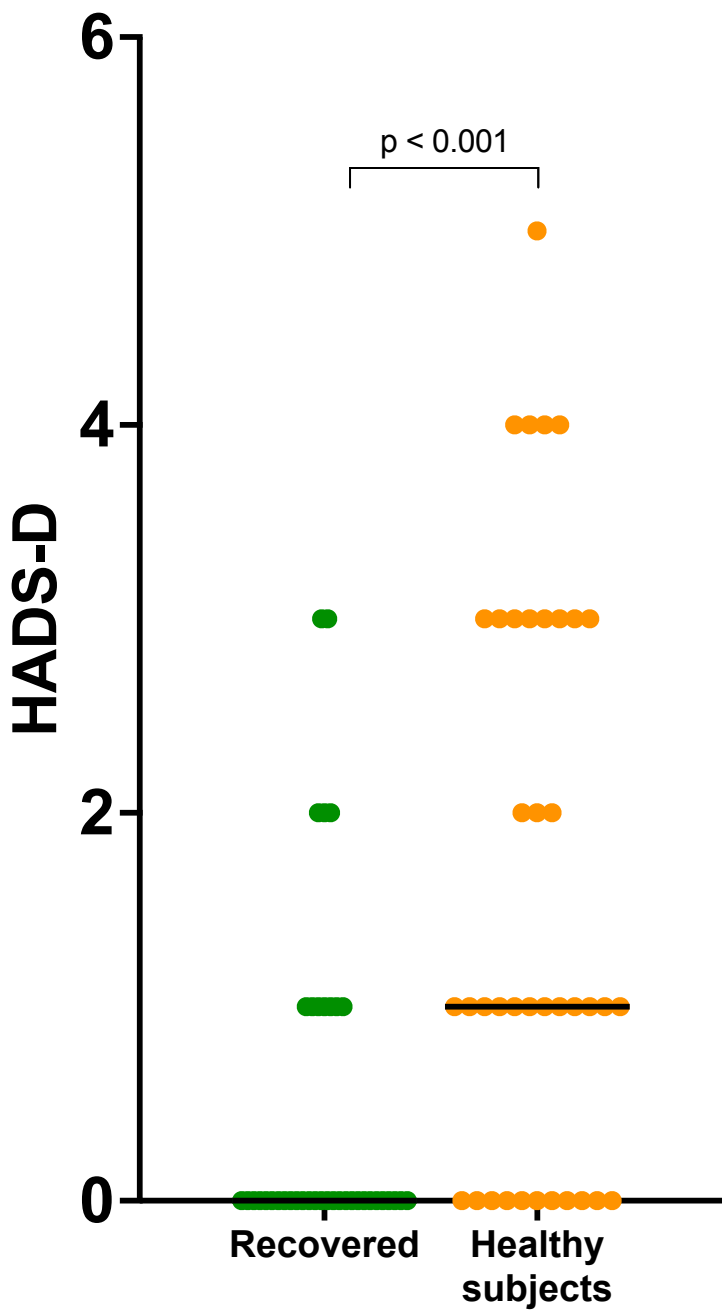

Supplement: Multimedia component 4 — Supplementary Figure 2. Comparison of HADS-D scores among recovered controls, and healthy pre-pandemic controls (n = 40 per group), with significantly higher HADS-D scores in healthy controls (p = 0.001). [file mmc4.pdf]
